# Dihydroartemisinin-sodium taurocholate-PLGA nanoparticles: a novel therapeutic approach against cystic echinococcosis

**DOI:** 10.3389/fphar.2025.1600525

**Published:** 2025-06-11

**Authors:** Aierpati Moheteer, Jiang Zhu, Dongming Pang, Xue Rao, Nijiati Aini, Kalibixiati Aimulajiang, Zhenping Zhang, Saifuding Abula, Adelijiang Wusiman

**Affiliations:** ^1^ College of Veterinary Medicine, Xinjiang Agricultural University, Urumqi, China; ^2^ Xinjiang Key Laboratory of New Drug Study and Creation for Herbivorous Animal (XJ-KLNDSCHA), College of Veterinary Medicine, Xinjiang Agricultural University, Urumqi, China; ^3^ Department of Abdominal Surgery, The Third People Hospital of Xinjiang Uygur Autonomous Region, Urumqi, China; ^4^ State Key Laboratory of Pathogenesis, Prevention and Treatment of High Incidence Diseases in Central Asia, Clinical Medicine Institute, The First Affiliated Hospital of Xinjiang Medical University, Urumqi, China

**Keywords:** Echinococcus granulosus, dihydroartemisinin, sodium taurocholate, PLGA, preparation of nanoparticles, treatment

## Abstract

**Introduction:**

Dihydroartemisinin (DHA) demonstrates potent anti-echinococcal activity. However, its clinical application is constrained by non-specific biodistribution and low bioavailability. To overcome these limitations and enhance hepatic targeting, the DHA was encapsulated in poly (lactic-co-glycolic acid) (PLGA) nanoparticles, and using sodium taurocholate (STC) as the surface modifier, a novel composite nanoparticle designated as DHA-STC-PLGA nanoparticles (DSP nanoparticles).

**Methods:**

The formulation was optimized using response surface methodology, and its targeting efficiency was confirmed through in-vivo fluorescence imaging. Both in vitro and in vivo studies were conducted to evaluate the therapeutic efficacy and elucidate the underlying mechanisms of DSP nanoparticles in a murine model of cystic echinococcosis.

**Results:**

The results showed that the optimal preparation conditions of DSP nanoparticles were 40 mg/mL STC, a DHA/PLGA1:10, ultrasonic power (UP) of 80, and oil in water (O/W) ratio was 1:20. Under these conditions, the DSP nanoparticles size were 125.73 ± 1.78 nm, exhibited a sustained release of DHA, and maintained stability for up to 42 days. DSP nanoparticles demonstrated good safety at a dosage of 200 mg/kg. Additionally, DSP nanoparticles demonstrated effective targeting of the liver and intestines. Therapeutic evaluation in a DSP nanoparticles-treated mouse liver hydatid model revealed that the DSP nanoparticles-H group significantly reduced liver, spleen, and vesicle weights compared to both control and albendazole (ABZ)-treated groups (*P* <0.05). Furthermore, the DSP nanoparticles-H group significantly decreased serum levels of aspartate aminotransferase (AST), alanine aminotransferase (ALT), total bilirubin (TBIL), direct bilirubin (DBIL), and alkaline phosphatase (ALP). Additionally, levels of interleukin-6 (IL-6), tumor necrosis factor-alpha (TNF-α), and interleukin-10 (IL-10) were significantly reduced in both serum and cystic fluid, while interferon-gamma (IFN-γ) levels were markedly increased (*P* <0.05). In vitro assays further demonstrated that DSP nanoparticles exert anti-echinococcal effects by compromising the integrity of the cyst wall. Mechanistic results suggest that DSP nanoparticles exert potent anti-echinococcal effects through activation of the Wnt signaling pathway and its key regulatory genes.

**Discussion:**

Overall, these findings indicate that DSP nanoparticles represent a promising liver-targeted nanoformulation that not only enhances DHA bioavailability but also offers a potent therapeutic strategy against cystic echinococcosis.

## 1 Introduction

Dihydroartemisinin (DHA), a derivative of artemisinin, has been extensively studied and shown to possess significant antiparasitic, antimalarial, antitumor, and anti-inflammatory activities (Huang et al., 2023; Li et al., 2024). Moreover, DHA exhibits strong inhibitory effects on cystic hepatic echinococcosis *in vitro* (Ma et al., 2020). Upon entering the body, DHA also demonstrates antiparasitic effects, as it can effectively inhibit the growth of hepatic *Echinococcus granulosus* (*E. granulosus*) within the host and mitigate its detrimental effects on host organs (Wen et al., 2023). However, DHA is limited by its poor water solubility, low bioavailability, susceptibility to oxidation, and rapid *in vivo* degradation, all of which contribute to a loss of its therapeutic activity (Wong et al., 2022).

Poly (lactic-co-glycolic acid) (PLGA) is a nanoparticle carrier material known for its excellent biocompatibility and safety. Water-in-oil nanoparticles formulated with PLGA can encapsulate substantial quantities of lipophilic drugs, thereby enhancing their bioavailability and extending their therapeutic effects through sustained release (Kumar et al., 2023). Consequently, encapsulating DHA within PLGA nanoparticles improves its stability, prolongs its *in vivo* circulation time, and enhances its bioavailability. Previous research demonstrated that DHA-encapsulated PLGA nanoparticles significantly prolonged the *in vivo* circulation time of DHA and notably its antimalarial efficacy (Zuo H et al., 2022). To further enhance the hepato-intestinal targeting of DHA-PLGA nanoparticles and boost their therapeutic effect against hepatic *E. granulosus*, sodium taurocholate (STC) was used as a surfactant. STC, a natural bile salt, not only improves drug encapsulation efficiency but also facilitates liver targeting by specifically interacting with bile acid transport proteins (Lv et al., 2025).

To develop liver-targeted DHA-STC-PLGA (DSP nanoparticles) with therapeutic efficacy against *E. granulosus*, this study first optimizes the preparation process of DSP nanoparticles using the response surface methodology (RSM) and evaluates their characterization, stability, and encapsulation efficiency. Subsequently, the safety of DSP nanoparticles was assessed by oral administration in mice, followed by an evaluation of their therapeutic effects in a mouse model infected with *E. granulosus*. Finally, *in vitro* experiments, including transcriptomic analysis and qPCR, were conducted to elucidate the antiparasitic mechanism of DSP nanoparticles.

## 2 Materials and methods

### 2.1 Preparation of DSP nanoparticles

To prepare DSP nanoparticles, 10 mg of DSP nanoparticles and 100 mg of PLGA were dissolved in 1 mL of chloroform to form the oil phase (O). Separately, 800 mg of STC was dissolved in 20 mL of ultrapure water to form the external aqueous phase (W). The oil phase was gradually added to the aqueous phase with continuous stirring to ensure uniform mixing. The resulting mixture was subjected to ultrasonic emulsification under ice-cooled conditions using a pulsed cycle mode (5 s activation/5 s rest) at 80% amplitude for 10 min to generate a thermodynamically stable oil-in-water (O/W) emulsion. The emulsion was then transferred to a rotary evaporator to remove chloroform under reduced pressure. The resulting dispersion was centrifuged at 3,000 rpm for 5 min to eliminate larger particles and impurities. The supernatant containing the DSP nanoparticles was collected and stored at 4°C for further use.

### 2.2 Optimization of DSP nanoparticles preparation conditions by single-factor experiments

Using the encapsulation efficiency of DHA as the evaluation index, a series of single-factor experiments were conducted to investigate the effects of various formulations and process parameters on the DHA encapsulation efficiency. The investigated parameters included: STC concentrations (0.1%, 0.2%, 0.4%, 0.8%, 1.6%), mass ratios of DHA to PLGA (1:7, 1:8, 1:9, 1:10, 1:11), volume ratios of the aqueous phase to the oil phase (O/W) (1:15, 1:20, 1:25, 1:30, 1:35), and ultrasonic power (UP) (60 W, 70 W, 80 W, 90 W, 100 W).

### 2.3 Optimization of DSP nanoparticles preparation conditions using response surface methodology

Based on the results of the single-factor experiments, STC concentration, the mass ratio of DHA to PLGA, and ultrasonic power (UP) were identified as critical factors. To further optimize these parameters, a three-factor, three-level response surface methodology (RSM) was designed using Design Expert 12 software (Table 1). A quadratic polynomial model was constructed to evaluate the interaction effects among the selected factors on DHA encapsulation efficiency. The significance of the model was confirmed through analysis of variance (ANOVA), and its predictive accuracy and reliability were validated by assessing the goodness-of-fit (R^2^ value), P-value, and lack-of-fit tests.

**TABLE 1:** Factors and levels of the response surface experiment.

Fact			
Level	A: STC(mg/mL)	B: DHA/PLGA	C: UP
−1	30	1:9	70
0	40	1:10	80
1	50	1:11	90

The designed response surface model equation is as follows: Y represents the predicted response, β_0_ denotes the intercept of the model, βi signifies the linear effect of each independent variable Xi, βii indicates the quadratic effect of each independent variable, and βij represents the interaction effect between variables. ϵ stands for the random error term, and k is the total number of factors included in the model (Khuri, 2017; Reji and Kumar, 2022). The adequacy of the predictive response function (Y) of the selected model was evaluated through analysis of variance (ANOVA) (Gu et al., 2018).

$$Y=\beta_{0}+\sum_{i=1}^{k}\beta_{i}X_{i}+\sum_{i=1}^{k}\beta_{ii}X_{i}^{2}+\sum_{i<j}\beta_{ij}X_{i}X_{j}+\epsilon$$

### 2.4 Characterization, encapsulation efficiency, and stability assessment of DSP nanoparticles

The morphology of the DSP nanoparticles was characterized using scanning electron microscopy (SEM) (Zeiss Supra55 VP, Germany). To determine the encapsulation efficiency, the prepared nanoparticle emulsion was subjected to low-speed centrifugation to separate the precipitate. The amount of unencapsulated DHA present in the supernatant was quantified using high-performance liquid chromatography (HPLC) (Agilent Technologies, Germany). The encapsulation efficiency (EE%) was calculated using the following formula:

$$\text{Encapsulation Efficiency }\left(\%\right)=\left[\left(\text{Total Drug Amount }–\text{ Unencapsulated Drug Amount}\right)\;/\text{ Total Drug Amount}\right]\times 100\%$$

The DSP nanoparticles samples were stored at a temperature of 4°C, and dynamic light scattering (DLS) was used to measure the particle size distribution, zeta potential, and polydispersity index (PDI) at designated time points: days 0, 7, 14, 21, 28, 35, and 42. The stability of the nanoparticles was assessed by monitoring changes in these parameters over the storage period.

### 2.5 Safety evaluation of DSP nanoparticles

Thirty female Kunming mice (weighing 20 ± 1 g) were acclimated for 7 days under standard laboratory conditions and then randomly assigned to six groups (n = 5 per group): a blank Control group (administered physiological saline) and five treatment groups receiving DSP nanoparticles at intragastric doses of 25, 50, 100, 200 and 400 mg/kg, respectively. Treatments were administered once daily by gavage for 14 consecutive days. On day 14, the mice were anesthetized with diethyl ether, and blood samples were collected via orbital puncture for serum preparation. Following blood collection, mice were subsequently euthanized by cervical dislocation. Heart, liver, spleen, kidney, and small intestine tissues were harvested for further evaluation. All animal experimental procedures were approved by the Animal Welfare and Ethics Committee of Xinjiang Agricultural University (Approval No: 2023011) and conducted following institutional ethical guidelines.

### 2.6 *In Vivo* fluorescence imaging of DSP nanoparticles

Healthy female Kunming strain mice (weighing 20 ± 1 g) were arbitrarily allocated to two groups (n = 24 per group): a Control group and a DSP nanoparticles group. Mice in the DSP nanoparticles group received 0.2 mL of Cy5.5-loaded DSP nanoparticles via oral gavage, while those in the Control group received an equivalent volume of a mixture containing Cy5.5 fluorescent marker and physiological saline through the same route. At 2, 4, 6, 8, 12 and 24 h post-administration, mice were anesthetized and imaged using a small animal *in vivo* imaging system (IVIS Spectrum, PerkinElmer, United States) to monitor the distribution and migration dynamics of the fluorescent signal. Following imaging at each time point, mice were euthanized, and liver and intestinal tissues were harvested for further analysis to evaluate the targeted distribution of the drug within these organs.

### 2.7 Establishment of the hepatic *E. granulosus* model and *in vivo* experimental grouping

A hepatic *E. granulosus* infection was established in Kunming mice (20 ± 1 g) via intraperitoneal injection of viable protoscoleces. After a 7-day acclimation, 5,000 sterile protoscoleces were administered intraperitoneally under aseptic conditions to induce infection. Following model establishment, the mice were randomly divided into five experimental groups (n = 10 per group): the Model group (administered 0.2 mL of physiological saline via oral gavage), the ABZ group (albendazole 50 mg/kg), the DHA group (dihydroartemisinin 50 mg/kg), the DSP-L group (low-dose DSP nanoparticles 50 mg/kg), and the DSP-H group (high-dose DSP nanoparticles 100 mg/kg). All treatments were administered once daily by oral gavage for 30 consecutive days. Throughout the experiment, mice were monitored daily for clinical signs, behavior, body weight, food intake, and water consumption. At the end of the experiment, the mice were anesthetized with diethyl ether, and blood samples were collected via orbital puncture for serum separation. The animals were then euthanized by cervical dislocation, and relevant organs and tissues were harvested for subsequent analysis.

### 2.8 Tissue histopathological sectioning analysis

Liver and intestinal tissues were fixed in 4% paraformaldehyde for 48 h, followed by dehydration through a graded alcohol series, and embedded in paraffin. Paraffin-embedded tissues were sectioned into 4–5 μm-thick slices using a microtome. The sections were subsequently deparaffinized with xylene, rehydrated through a graded alcohol series, and stained with hematoxylin for 5–10 min. After rinsing under running water, the sections were differentiated with acid alcohol, blued with an alkaline buffer, and counterstained with eosin for 1–3 min. Finally, H&E-stained slides were examined using a light microscope for histopathological evaluation. For Masson staining, the deparaffinized and rehydrated sections were sequentially stained with Weigert’s iron hematoxylin for 10 min, acid fuchsin for 5–10 min, treated with phosphomolybdic acid for 5 min, and stained with aniline blue for 5 min. After dehydration through a graded alcohol series and mounting, the Masson-stained slides were observed under a microscope to evaluate collagen fiber distribution.

### 2.9 Determination of biochemical parameters and cytokines

An automated biochemical analyzer was utilized to measure serum concentrations of Alanine aminotransferase (ALT), Aspartate aminotransferase (AST), Total bilirubin (TBIL), Direct bilirubin (DBIL), Albumin (ALB), and Alkaline phosphatase (ALP). Enzyme-linked immunosorbent assay (ELISA) kits (Mouse ELISA Kit, Shfksc Company, China) were employed to quantify the levels of IL-6, TNF-α, IFN-γ, and IL-10 in both serum and cyst fluid. Standards and working solutions were prepared. As illustrated in the manufacturer’s instructions, samples along with standards were added to the ELISA plate wells for incubation. Following washing steps, the enzyme conjugate was introduced, followed by the substrate solution for color development. Absorbance was measured at the specified wavelength using a microplate reader, and analyte concentrations were determined based on the standard curve.

### 2.10 *In Vitro* anti-*echinococcal* activity of DSP nanoparticles

Protoscoleces with a viability of ≥95% were counted, and 200 viable protoscoleces were seeded per well in a 96-well plate. The wells were allocated into four groups: a blank Control group, an albendazole (ABZ) group, a dihydroartemisinin (DHA) group, and two DSP nanoparticles groups at low (DSP-L) and high doses (DSP-H). The drug concentrations for ABZ and DHA were set at 500 μmol/L and 250 μmol/L, respectively, while the DSP nanoparticles solutions were prepared at gradient concentrations of 1,400, 700, 350, 175, and 88 μmol/L. Cells were cultured at a temperature of 37°C. in a 5% CO_2_ incubator for 5 days. A dose-response curve was fitted using nonlinear regression to calculate the IC_50_ value. Viability of the protoscoleces was assessed daily using erythrosin staining, where red-stained specimens were considered dead or less viable, while unstained ones were deemed viable. Mortality rates were calculated based on observations under an inverted microscope. Protoscoleces from the Control group and DSP nanoparticles groups were collected, fixed in 50% FAA for 24 h, dehydrated through graded ethanol (30%–100%), placed in tert-butanol, freeze-dried, sputter-coated with gold, and then examined using SEM to observe ultrastructural changes.

### 2.11 Transcriptome sequencing of DSP nanoparticles Anti-E. *granulosus* activity

Before the experiment, fine-granular *E. granulosus* protoscoleces were isolated and cultured under sterile conditions to ensure a viability of at least 90%. The experiment was divided into two groups: the DSP nanoparticles group and the Control group, each with six biological replicates. Protoscoleces in the DSP nanoparticles group were exposed to nanoparticles for 48 h, whereas those in the Control group were incubated with drug-free medium under identical conditions for the same duration. Following treatment, the protoscoleces were collected and rinsed three times using PBS to eliminate residual drugs and medium, then rapidly frozen in liquid nitrogen. Total RNA extraction (Total RNA Isolation Reagent, Biosharp company, China) was performed as illustrated in the manufacturer’s protocol. After assessing RNA quality and concentration, a sequencing library was constructed. High-throughput sequencing was conducted, followed by quality Control of the transcriptome data, differential gene expression analysis, functional annotation, and gene enrichment analysis.

### 2.12 qRT-PCR validation of the main pathways

The extracted RNA was reverse transcribed into cDNA using a Reverse transcription kit (CellTotal RNA Isolation Kit, Fore Gene, China) as illustrated in the manufacturer’s protocol. Specific primers for the target genes and the reference gene (β-actin) were designed and synthesized (Table 2). Quantitative real-time PCR (qRT-PCR) (ChamQ SYBR qPCR Master Mix, Vazyme Biotech Co., Ltd., China) amplification was conducted using SYBR Green dye, with the reaction mixture and cycling conditions strictly adhering to the kit instructions. The amplification process was monitored in real time, and the specificity of the products was confirmed by analyzing the melting curve. The relative expression levels of the target genes were quantified using the 2^-ΔΔCt method.

**TABLE 2:** Primer’s sequence of qPCR.

Gene	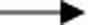 Primer sequence (5′-3′)	Base number
β-actin	AGC​CCC​CGT​ACA​ATC​CAA​AG	20
	GGT​AAC​ACC​GTC​ACC​GGA​AT	20
FRP	CAT​CGA​CAG​ACC​GAC​CTA​CG	20
	GAG​TGT​GGG​CAC​AAG​ATC​CA	20
Wnt	GGC​CCG​CCT​ACT​CTA​CAA​TC	20
	TGG​CGG​TGT​TTC​GTA​GTT​CA	20
CK2	GCC​GTC​TTC​CAT​CGG​TTT​TG	20
	GAA​TCC​ACG​ACA​CCT​CCT​CG	20
β-catenin	GGC​TGA​CTG​TCT​GTG​CTG​AT	20
	TTT​CCG​CAA​TGT​CGC​AAT​CC	20
TAK1	CCG​ATA​GAC​CTG​TCC​CCA​GA	20
	TTT​CCA​CGG​TAT​GTG​CCG​AA	20
CKIα	ATT​GGG​GCT​GGA​TCT​TTC​GG	20
	TCG​AGG​AAC​GGC​AAA​AGT​GT	20
SIRT1	GCA​GAA​ATC​CTC​CTG​TGG​CT	20
	TCC​TTC​GTC​TCT​GTC​TCC​GT	20
Pontin52	TAC​GGG​GAC​TGG​GAT​TGG​AT	20
	GCC​ATT​AAA​ACA​GCC​CGA​CC	20
CBP	GGG​TCT​TCC​TCC​TCA​GCA​AC	20
	TAC​AAG​GCG​TGG​AAT​GGG​TC	20

### 2.13 Data statistics and analysis

Experimental results are expressed as mean ± standard deviation (SD). Dose-response relationships were analyzed by nonlinear regression to determine IC_50_ values using a four-parameter logistic model in GraphPad Prism 8.0. Statistical significance was established at *P* < 0.05, with intergroup comparisons performed via one-way ANOVA followed by Tukey’s post-hoc test for multiple comparisons, SPSS 26.0. Response surface methodology and optimization modeling were implemented in Design Expert 12. Graphical representations were created using GraphPad Prism 8.0.

## 3 Results and discussion

### 3.1 Results of the single-factor experiments

The formation and encapsulation efficiency of nanoparticles are influenced by several key factors, including surfactant concentration, drug-to-PLGA ratio, oil-to-water ratio, and ultrasonic power (Wusiman et al., 2022a). Among these, STC, acting as a surfactant, plays a pivotal role in enhancing nanoparticles’ stability and drug distribution (Zhang C et al., 2021). The ratio of DHA/PLGA significantly affects the drug-loading capacity and structural stability of the nanoparticles (Tran et al., 2024; Zuo H. et al., 2022). The oil-in-water (O/W) ratio, defined as the volume ratio of the oil phase to the water phase, significantly influences emulsification efficiency and nanoparticles formation (Liu et al., 2023). Ultrasonic power is essential for emulsification and precise Control of particle size during nanoparticles preparation (Catania et al., 2022;Sreeharsha et al., 2024).

**FIGURE 1:**
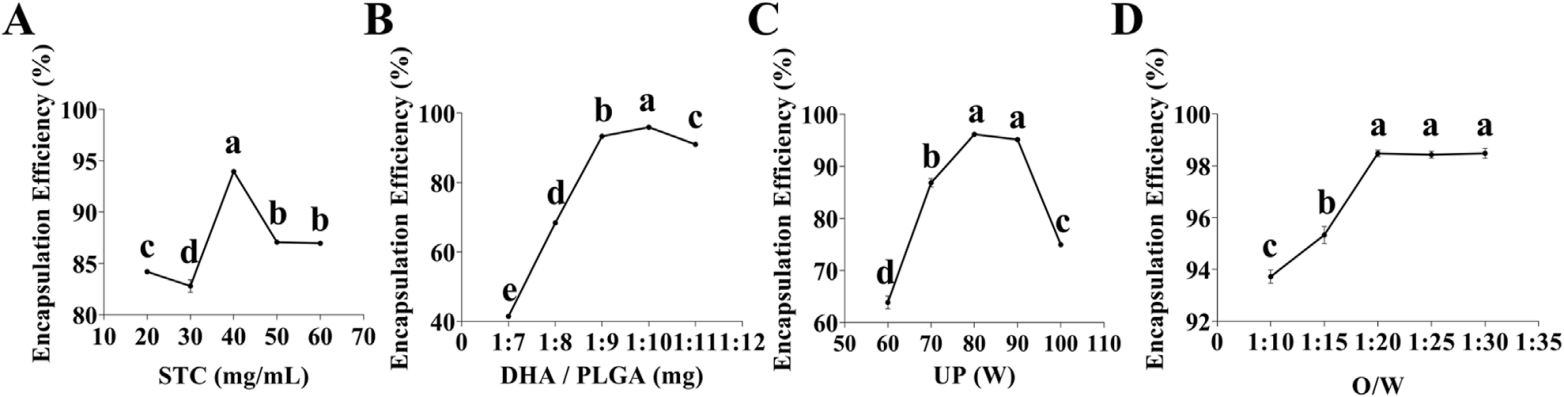
Single-factor test. **(A)** Effect of STC concentration on DHA encapsulation efficiency (n = 3), **(B)** Effect of DHA to PLGA mass ratio on DHA encapsulation efficiency (n = 3), **(C)** Effect of UP on DHA encapsulation efficiency (n = 3), **(D)** Effect of O/W ratio on DHA encapsulation efficiency (n = 3). Lowercase letters a, b, c and d indicates significant differences (*P* < 0.05).

As illustrated in Figure 1A, the optimal concentration of STC was determined to be 40 mg/mL. Concentrations that are either excessively high or low can lead to over-stabilization of the emulsion or the formation of loosely structured nanoparticles, thereby reducing encapsulation efficiency. Similarly, as shown in Figure 1B that the optimal DHA/PLGA ratio was identified as 1:10. Deviations from this ratio negatively affect the encapsulation capacity of PLGA nanoparticles, leading to partial DHA precipitation into the aqueous phase and reduced overall encapsulation efficiency. As illustrated in Figure 1C, the optimal ultrasonic power (UP) was 80 W. Excessive UP can damage the structural integrity of the nanoparticle, whereas insufficient UP can lead to inadequate nanoparticle formation, drug leakage, and poor dispersion, all of which lower encapsulation efficiency. As shown in Figure 1D, the highest encapsulation efficiency was achieved at an O/W ratio of 1:20. Ratios exceeding 1:20 do not significantly affect encapsulation efficiency. These findings indicate that the nanoparticles system achieves optimal stability and encapsulation efficiency under the following conditions: STC concentration of 40 mg/mL, the DHA/PLGA ratio of 1:10, UP is 80 W, and the O/W ratio of 1:20. During the nanoparticles preparation process, the STC concentration, DHA/PLGA ratio, and UP play crucial roles in determining structural stability and drug encapsulation efficiency.

### 3.2 Results of the response surface

Response Surface Methodology (RSM) is extensively used for the optimization of experimental processes involving multiple factors and levels. In the context of nanoparticles preparation, RSM facilitates the systematic design of experiments and the development of a predictive mathematical model that links process parameters with response values. This approach enables the determination of optimal process conditions by systematically evaluating both the main effects and interactions of the factors (Wusiman A et al., 2022b). As shown in Table 3, Design-Expert 12 software was utilized to perform response surface optimization for factors influencing the encapsulation efficiency of DHA in nanoparticles, specifically, (A) STC, (B) DHA/PLGA ratio, and (C) UP. Based on a comparison between the predicted and observed values under 17 experimental conditions, the following regression equation was derived: Y = 98.47 + 6.00A + 0.1858B + 1.29C - 0.6925AB - 0.5545AC - 0.2947BC - 4.00A^2^ - 2.82B^2^ - 3.33C^2^. The three-dimensional response surface plots depict the influence of individual factors on the response.

**TABLE 3:** Response surface experimental design and results.

Run order	Factor			Encapsulation efficiency	
	A	B	C	Actual value	predicted value
1	1	1	0	97.82	97.14
2	−1	0	1	87.09	86.98
3	0	−1	−1	91.04	90.55
4	−1	−1	0	84.09	84.77
5	0	0	0	98.01	98.47
6	1	0	−1	96.3	96.41
7	0	0	0	98.03	98.47
8	0	0	0	99.05	98.47
9	0	0	0	98.34	98.47
10	0	1	1	93.03	93.51
11	−1	1	0	86.9	86.52
12	0	1	−1	90.94	91.51
13	1	0	1	97.68	97.88
14	−1	0	−1	83.49	83.29
15	1	−1	0	97.78	98.16
16	0	−1	1	94.3	93.73
17	0	0	0	98.95	98.47

As illustrated in Table 4, the regression analysis of the model demonstrated a high level of significance (*P* < 0.0001), with a correlation coefficient R^2^ = 0.9928 and an adjusted R^2^ = 0.9835, both approaching 1.000. The coefficient of variation (CV) was 0.7461%. The F-value analysis showed that the degree of influence on the encapsulation efficiency of DSP nanoparticles was STC (A) > UP (C) > DHA/PLGA(B). These results indicate that the optimal extraction conditions for DSP nanoparticles were as follows: STC concentration of 40 mg/mL, DHA/PLGA ratio of 1:10, ultrasonic power (UP) of 80 W, and oil/water (O/W) phase ratio of 1:20. These parameters collectively yielded an actual DHA content of 99.05% with a closely aligned predicted value of 98.47%. The close agreement between the predicted and measured values indicates that the regression model provides an excellent fit to the data and is suitable for optimizing the actual nanoparticle formulation process, thereby validating its accuracy and applicability.

**TABLE 4:** Analysis of variance (ANOVA) for the regression model.

Source	Sum of squares	df	Mean square	F-value	p-value	
Model	469.94	9	52.22	106.84	<0.0001	significant
A- STC	288.29	1	288.29	589.89	<0.0001	
B-DHA/PLGA	0.2761	1	0.2761	0.5649	0.4768	
C-UP	13.34	1	13.34	27.30	0.0012	
AB	1.92	1	1.92	3.92	0.0880	
AC	1.23	1	1.23	2.52	0.1567	
BC	0.3475	1	0.3475	0.7111	0.4270	
A^2^	67.52	1	67.52	138.16	<0.0001	
B^2^	33.51	1	33.51	68.56	<0.0001	
C^2^	46.63	1	46.63	95.41	<0.0001	
Residual	3.42	7	0.4887			
Lack of Fit	2.44	3	0.8124	3.30	0.1393	not significant
Pure Error	0.9837	4	0.2459			
Cor Total	473.36	16				

### 3.3 Characterization, encapsulation, and stability of DSP nanoparticles

**FIGURE 2:**
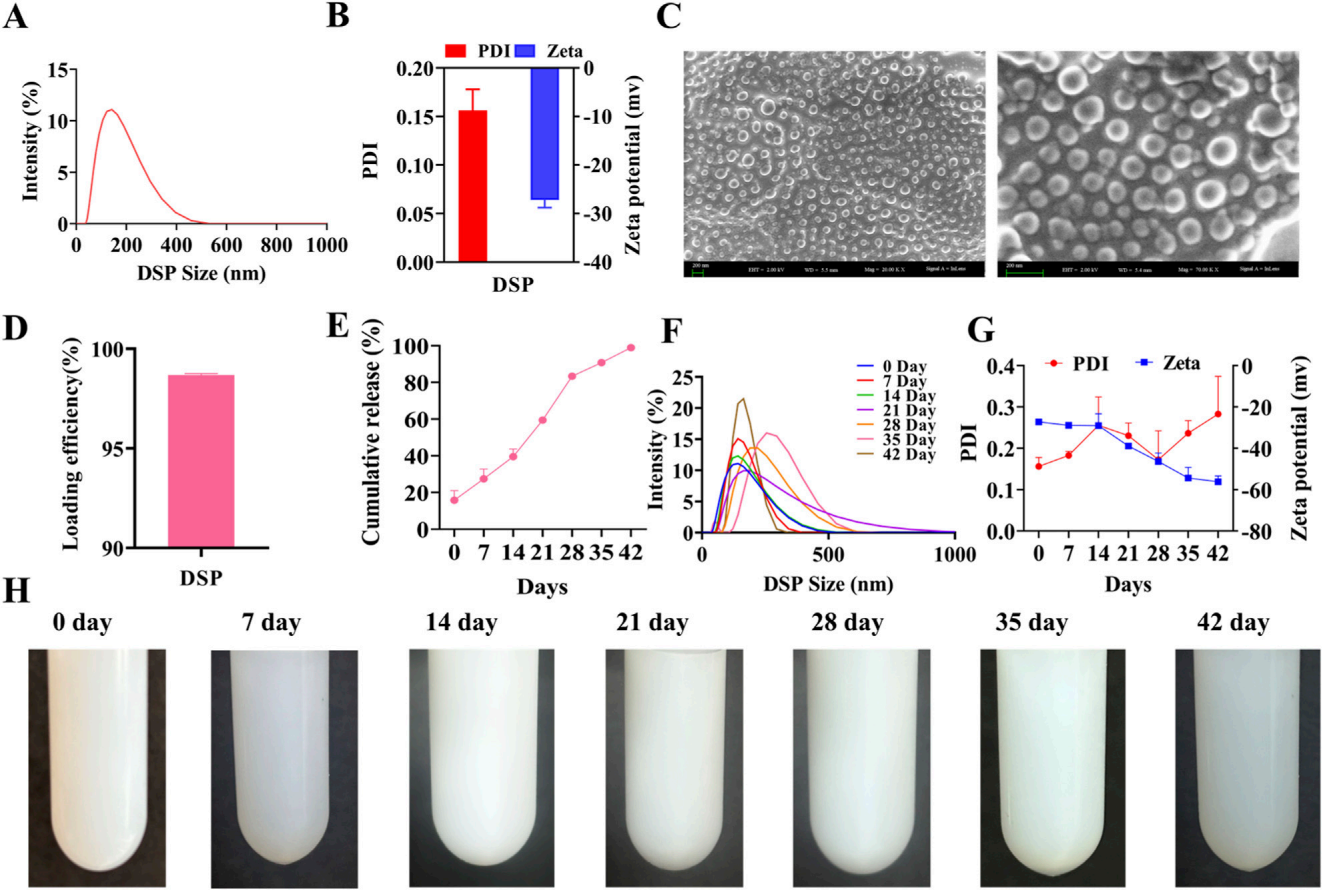
Characterization, Encapsulation and Stability of DSP nanoparticles. **(A)** Nanoparticle’s size (n = 3). **(B)** Nanoparticles zeta potential and nanoparticles PDI (n = 3). **(C)** Nanoparticles SEM. The particles are spherical with a uniform size distribution below 200 nm. Scale bar = 200 nm (n = 3). **(D)** Nanoparticle’s encapsulation efficiency (n = 3). **(E)** *In vitro* nanoparticles release (n = 3). **(F)** Nanoparticles characterization (n = 3). **(G)** Nanoparticles PDI and Zeta Potential at 42 Days (n = 3). **(H)** Stability of nanoparticles (n = 3).

The characterization, encapsulation efficiency, and stability of nanoparticles are critical factors that determine the efficacy of a drug delivery system. High-resolution SEM and dynamic light scattering (DLS) are widely used to obtain precise measurements of the nanoparticle morphology and size distribution (Bals and Epple, 2023; Guerrero-Florez et al., 2024). As illustrated in Figures 2A,B, the average particle size of DSP nanoparticles was 125.73 ± 1.78 nm, with a zeta potential of −27.23 ± 1.54 mV and a polydispersity index (PDI) of 0.16 ± 0.02. These values indicate that the nanoparticles were small, uniformly distributed, and exhibited a stable negatively charged surface. As shown in Figure 2C, SEM revealed that the nanoparticles possessed smooth, spherical morphology with a consistent size distribution below 200 nm.

Encapsulation efficiency is a critical parameter for evaluating the drug loading capacity of nanoparticle-based delivery systems, as it directly impacts their therapeutic efficacy and safety. A higher encapsulation efficiency enhances drug utilization and contributes to improving sustained-release performance. This dual benefit enables reduced dosing frequency while minimizing systemic side effects, ultimately contributing to more effective therapeutic outcomes (Marques and Segundo, 2024). As illustrated in Figure 2D, high-performance liquid chromatography (HPLC) revealed an encapsulation efficiency of 98.68% ± 0.07%, indicating highly efficient drug encapsulation (see Supplementary Figure S2 for the HPLC chromatogram). Furthermore, as shown in Figure 2E, the DSP nanoparticles exhibited a sustained release profile over 42 days, confirming their prolonged drug delivery capability.

A stable nanoparticle system is essential for maintaining optimal particle size and encapsulation efficiency during long-term storage, ensuring consistent and controlled groupable drug release upon application (Fernando et al., 2019). As illustrated in Figures 2F,G, a gradual increase in particle size and PDI was observed over time, but remained below 0.3, with satisfactory stability. Additionally, Figure 2H confirms that no aggregation or precipitation occurs within 35 days, further supporting the excellent stability of the nanoparticles. Collectively, these findings indicate that DSP nanoparticles possess favorable characteristics, including optimal particle size, morphology, encapsulation efficiency, sustained-release performance, and long-term stability.

### 3.4 Safety evaluation of DSP nanoparticles

The murine safety assessment is designed to evaluate the systemic toxicity of a drug and detect any organ-specific toxicities, thereby establishing a foundation for subsequent clinical trials. Orally administered drugs are absorbed through the intestinal tract and enter the portal venous system, where they initially undergo hepatic metabolism before reaching systemic circulation (Herman and Santos, 2023).

Therefore, assessing the toxicity of the drug on vital organs such as the heart, liver, spleen, kidneys, and small intestine is an essential prerequisite for evaluating its pharmacological activity (Ali and Jihad, 2021). Following 14 days of oral gavage administration, no significant abnormalities in behavior or food intake were observed in either the control group or experimental groups, and no mortality occurred. H&E-stained sections of the heart, liver, spleen, kidneys, and small intestine (Supplementary Figure S3) revealed that the tissue architecture in both the DSP nanoparticles-treated and control groups was normal, with no evident pathological changes. As illustrated in Table 5, at DSP nanoparticle concentrations of 200 mg/kg or lower, all measured biochemical parameters remained within the normal reference ranges. However, at a concentration of 400 mg/kg, TBIL and UREA levels exceeded the normal reference ranges. These findings suggest that DSP nanoparticles do not induce significant adverse effects on the heart, liver, spleen, kidneys, or intestines at concentrations of 200 mg/kg or lower, supporting their safety at therapeutic doses.

**TABLE 5:** Serum biochemical indicators in mice.

Index	ALT (U/L)	AST (U/L)	TBIL (umol/L)	DBIL (umol/L)	UREA (mmol/L)	CREA (umol/L)
Normal	10.06–96.47	36.31–235.48	6.09–53.06	0.45–33.89	3.9–12.4	10.91–85.09
Control group	25.55 ± 3.08	110.22 ± 22.45	33.29 ± 17.08	10.41 ± 2.32	5.73 ± 0.45	25.58 ± 2.92
400 mg/kg	41.98 ± 0.88	188.06 ± 18.25	88.75 ± 31.46	19.45 ± 2.69	26.89 ± 2.81	14.04 ± 0.54
200 mg/kg	35.62 ± 1.15	145.78 ± 17.77	36.48 ± 9.43	14.58 ± 2.17	7.08 ± 0.35	17.02 ± 1.34
100 mg/kg	32.04 ± 1.03	137.58 ± 13.75	34.83 ± 23.93	8.89 ± 4.09	6.92 ± 0.69	22.38 ± 1.25
50 mg/kg	30.5 ± 1.94	118.55 ± 34.93	34.31 ± 14.68	8.99 ± 3.05	8.44 ± 0.44	18.97 ± 2.5
25 mg/kg	24.89 ± 2.68	113.2 ± 15.16	34.83 ± 14.79	7.45 ± 0.85	8.16 ± 0.31	22.46 ± 3.68

### 3.5 *In Vivo* fluorescence imaging detection

**FIGURE 3:**
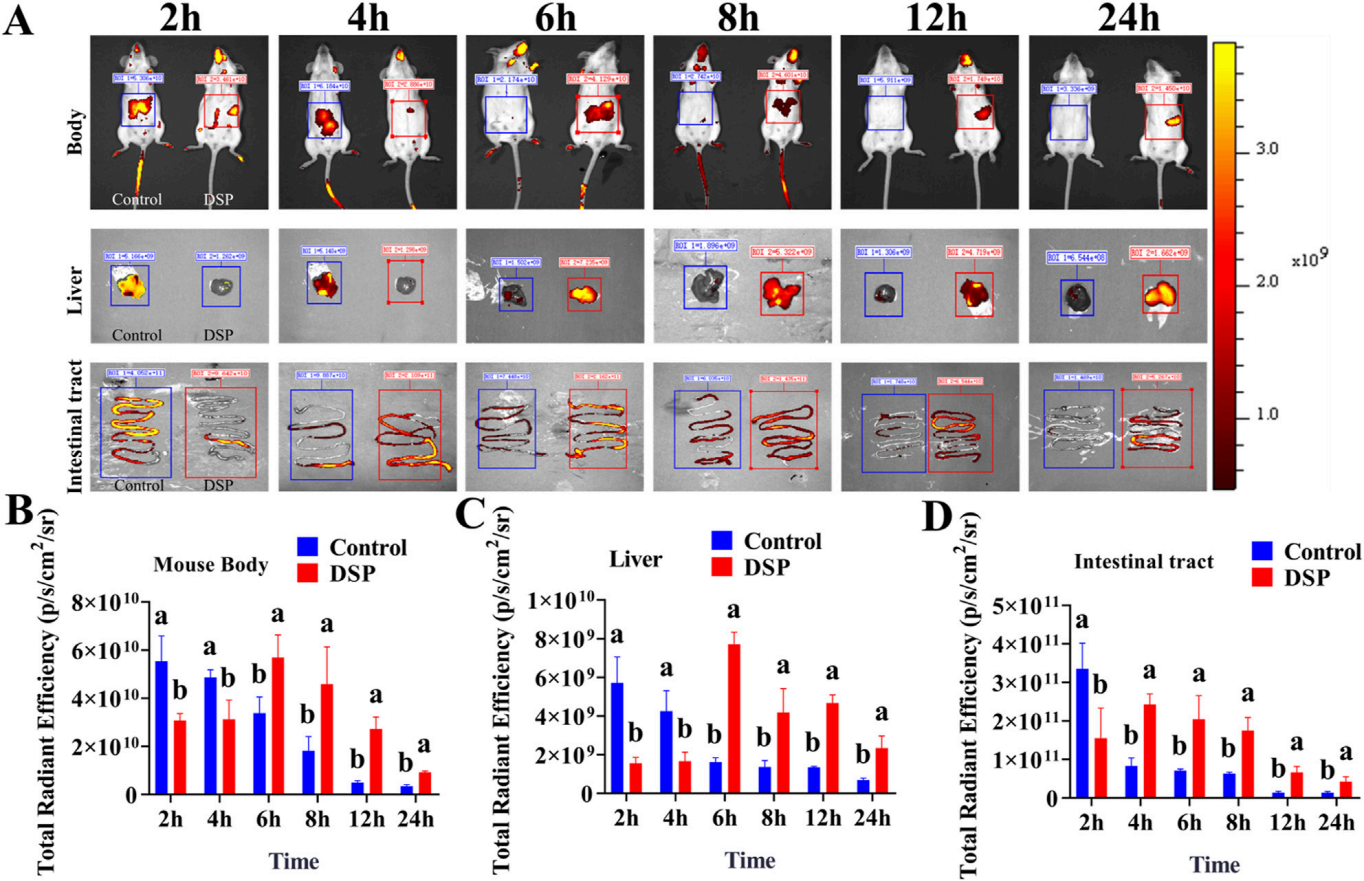
Fluorescence imaging of whole body, liver, and intestine in mice. **(A)** *In vivo* fluorescence imaging of the whole body, liver, and intestine in mice (n = 4), **(B)** Whole-body fluorescence index (n = 3), **(C)** Liver fluorescence index (n = 3), **(D)** Intestinal fluorescence index (n = 3). Lowercase letters a, b indicates significant differences (*P* < 0.05).

By quantitatively analyzing the intensity and temporal dynamics of fluorescence signals, the concentration of the probe in target regions (e.g., liver or intestine) can be assessed. This analysis facilitates the evaluation of drug accumulation, clearance rate, and targeting efficiency (Wang S et al., 2016). As illustrated in Figure 3A, fluorescence signals were recorded at 2, 4, 6, 8, 12, and 24 post-oral administration of the fluorescence-labeled control group and DSP nanoparticles. An *in vivo* fluorescence imaging system was used to determine their distribution dynamics in the whole body, liver, and intestine. As illustrated in Figures 3B–D, the fluorescence signal intensity in the whole body, liver, and intestine of the control group showed a gradual decreasing trend over time. In contrast, the DSP group showed an initial increase followed by a subsequent decrease in fluorescence intensity. As illustrated in Figures 3B,C, within 2–4 h, the control group exhibited significantly stronger whole-body and liver fluorescence signals compared to the DSP group (*P* < 0.05), and significantly lower than that in the DSP group at 6–24 h (*P* < 0.05). The results show that DSP nanoparticles have significant whole-body and liver targeting properties. As illustrated in Figure 3D, the fluorescence intensity of the control group at 2 h was significantly higher than that of DSP group (*P* < 0.05), and at 4–24 h was significantly lower than that of DSP group (*P* < 0.05), indicating that DSP nanoparticles had significant intestinal retention characteristics.

### 3.6 Effects of DSP nanoparticles on the mouse hepatic *E. granulosus* model

**FIGURE 4:**
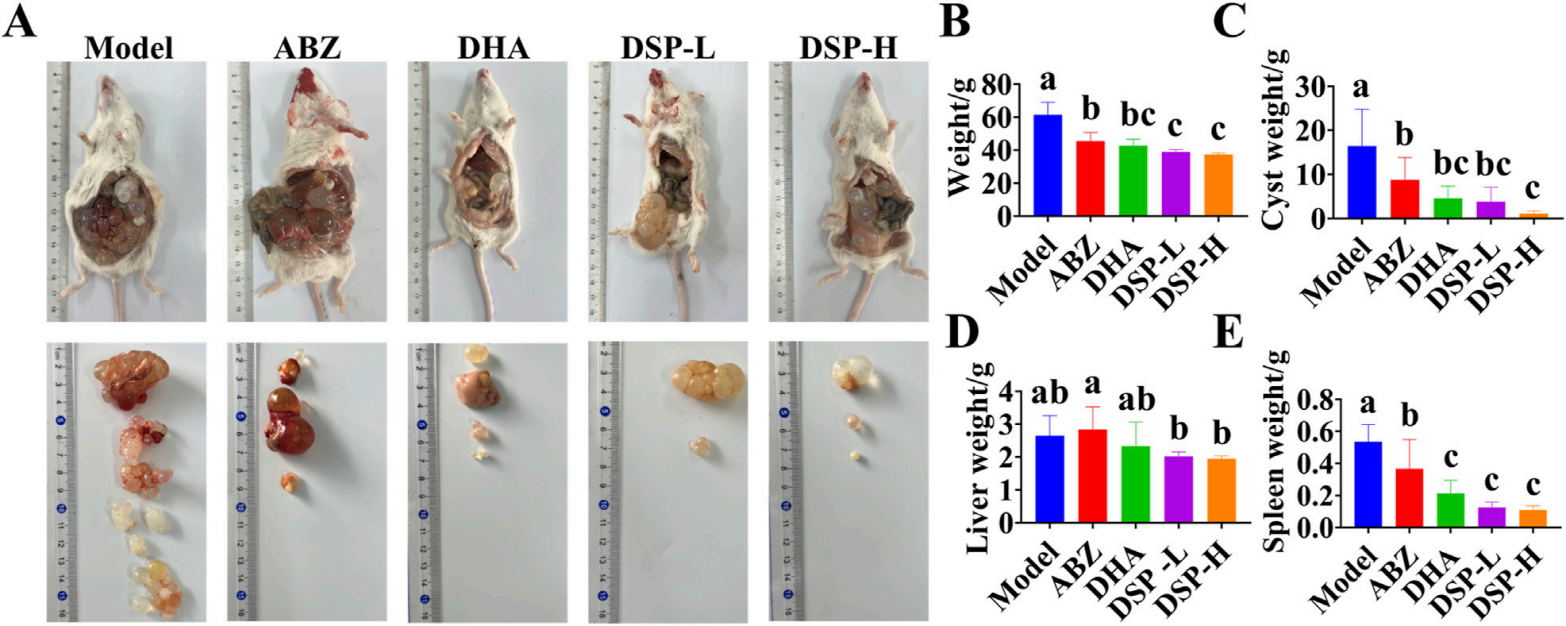
Establishment and treatment of mouse model of hepatic hydatid disease. **(A)** Effect of DSP nanoparticles on a murine hepatic echinococcosis model (n = 5), **(B)** Mouse body mass index (n = 5), **(C)** Vesicle mass index (n = 5), **(D)** Liver mass index (n = 5), **(E)** Spleen mass index (n = 5). Lowercase letters a, b, c indicates significant differences (*P* < 0.05).

Echinococcal vesicles represent the primary pathological hallmark of hepatic echinococcosis. And their weight provides a direct and reliable indicator for assessing the severity of infection (Gessese, 2020). The liver, functioning as the main blood filtration organ, is the most common site of Echinococcal cysts development as *E. granulosus* eggs are transported through the bloodstream and preferentially localize in hepatic tissue. Therefore, liver weight can serve as a reliable indicator for evaluating the extent of hepatic echinococcosis infection (Wen et al., 2019). The spleen serves as a secondary target organ, may also be affected in *granulosus* infection. When cysts originating in the liver can disseminate through the bloodstream to the spleen, potentially leading to splenomegaly and associated abdominal discomfort (Liu et al., 2022). As illustrated in Figure 4A, the ABZ, DHA, DSP-L, and DSP-H treatment groups exhibited reduced cyst volumes compared to the model group, with the smallest cysts in the DSP-H group showing shrinkage and clear cyst fluid. As illustrated in Figures 4B,C, the ABZ, DHA, DSP-L, and DSP-H groups showed significant decreases in body weight index and cyst weight index compared to the Model group (*P* < 0.05). Additionally, the DSP-L and DSP-H groups showed significantly lower body weight indices in were significantly lower than those in the Model and ABZ groups (*P* < 0.05). The vesicle weight index in the DSP-H group was significantly lower weight indices than both the Model and ABZ groups (*P* < 0.05). As shown in Figures 4D,E, liver and spleen indices were significantly decreased in the DSP-L and DSP-H groups compared to the model group (*P* < 0.05). These findings suggest that DSP nanoparticles can effectively suppress cyst proliferation and mitigate organ pathology associated with hepatic *echinococcosis*.

### 3.7 H&E and masson staining of liver and intestine in the mouse

**FIGURE 5:**
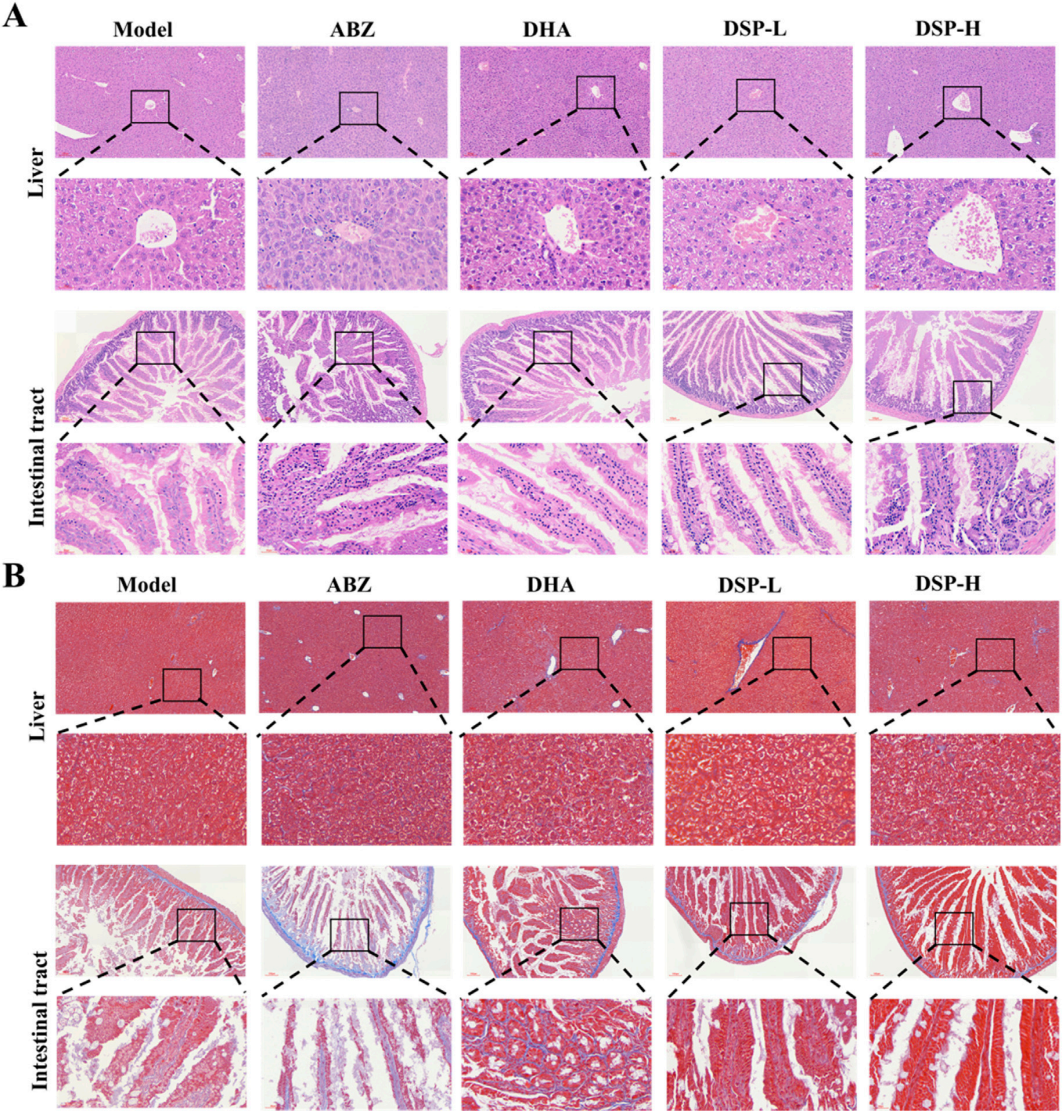
The liver and intestine of mice were stained with HE and Masson staining. **(A)** Liver and intestinal Hematoxylin and eosin (H&E) staining, **(B)** Liver and intestinal Masson’s trichrome staining.

Medical treatment of *E. granulosus* mainly acts through hepato-intestinal circulation, which may result in hepatic or intestinal damage (Ding et al., 2020). Hematoxylin and eosin (H&E) staining provides clear visualization of neutrophil and lymphocyte infiltration, as well as necrotic areas, serving as a crucial reference for assessment of organ inflammation and tissue injury (Yang et al., 2011). Masson staining, by specific labeling of collagen fibers, enables both quantitative and qualitative analysis of hepatic and intestinal fibrosis due to abnormal tissue repair processes (Luo et al., 2023). As illustrated in Figure 5A, H&E staining demonstrates that liver and intestinal tissues in the DSP nanoparticles-treated group exhibit intact structural integrity, with well-defined hepatic lobular architecture, and orderly arranged hepatocytes. No significant evidence of liver damage, cellular necrosis, or inflammatory infiltration was observed. The intestinal tissues exhibit delineated layers, without any apparent inflammatory response or tissue damage, and no significant differences compared to the Control group. Similarly, as illustrated in Figure 5B, Masson staining results indicate no obvious deposition of collagen fibers in the liver or intestinal tissues. The tissue structures remain normal, with no signs of fibrosis or other pathological changes relative to the control group. These findings suggest that, under the experimental conditions, DSP nanoparticles do not induce significant pathological damage or fibrotic responses in liver and intestinal tissues.

### 3.8 Analysis of serum biochemical indicators in mouse models *in vivo*

**FIGURE 6:**
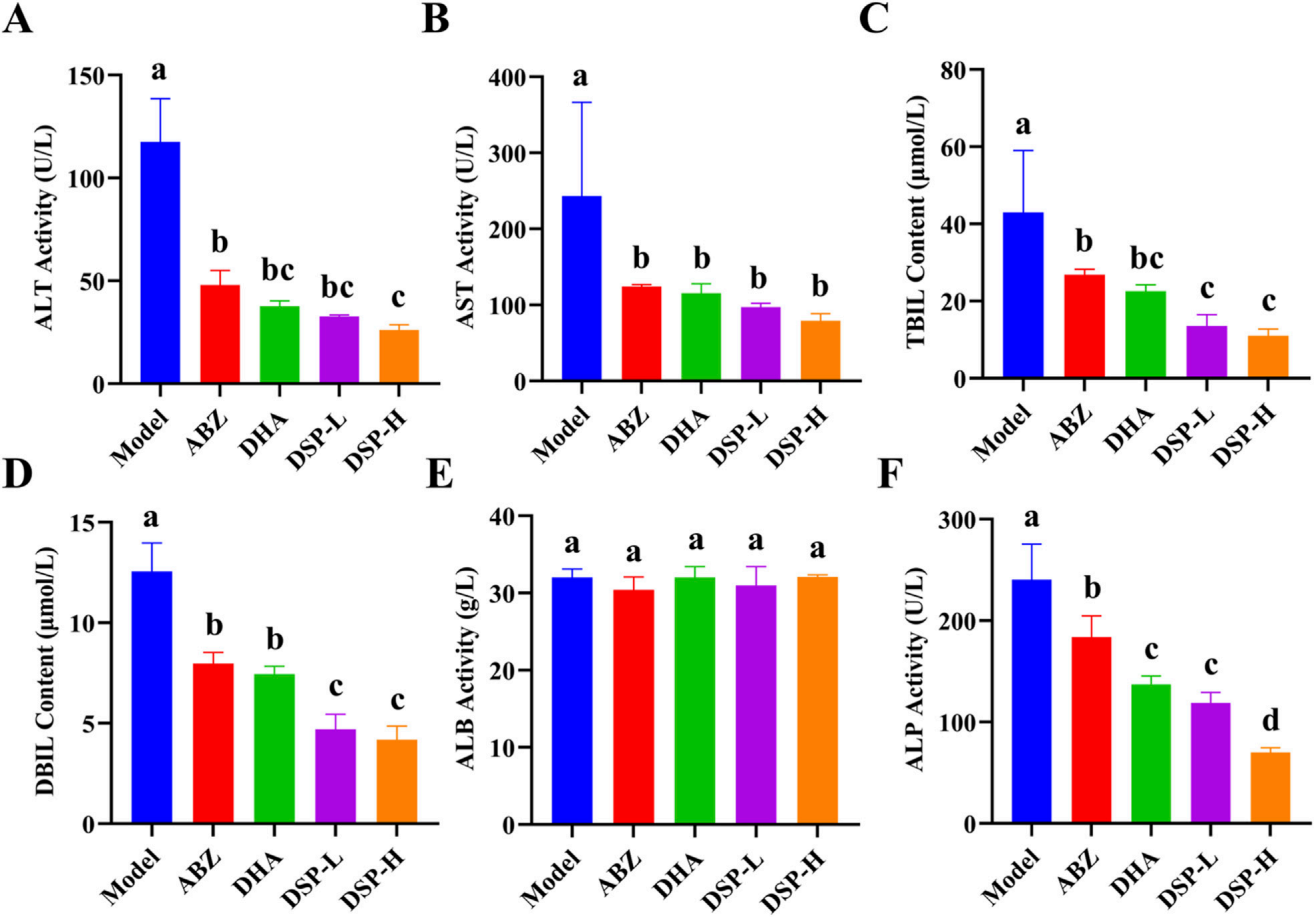
Analysis of serum biochemical indicators. **(A)** ALT Activity (n = 4), **(B)** AST Activity (n = 4), **(C)** TBIL Activity (n = 4), **(D)** DBIL Activity (n = 4), **(E)** ALB Activity (n = 4), **(F)** ALP Activity (n = 4). Lowercase letters a, b, c, and d indicate significant differences (*P* < 0.05).

Serum biochemical indicators, including ALT, AST, TBIL, and DBIL, are crucial for assessing liver function and detecting hepatic damage. These biomarkers provide essential references for evaluating hepatic status. ALP, which is predominantly present in the liver, bones, kidneys, and biliary system, serves as an important indicator of hepatobiliary function, with elevated levels suggesting potential abnormalities (Ali and Jihad, 2021). Albumin (ALB) is a vital protein synthesized by the liver that plays a critical role in maintaining plasma colloid osmotic pressure, thereby preventing fluid leakage from blood vessels into the interstitial spaces and thus avoiding edema (Luo et al., 2024). As illustrated in Figures 6A–F, the ABZ, DHA, DSP-L, and DSP-H treatment groups exhibited significant reductions in serum levels of AST, ALT, TBIL, DBIL, and ALP compared to the model group (*P* < 0.05). Moreover, the DSP-H group showed markedly decreased TBIL, DBIL, and ALP levels than both the ABZ and DHA groups (*P* < 0.05). Notably, ALB levels remained stable across all experimental groups (Figure 6E). These findings suggest that ABZ, DHA, DSP-L, and DSP-H can effectively alleviate liver dysfunction, with DSP-H exhibiting a particularly pronounced protective effect against hepatic injury.

### 3.9 Detection of cytokines in serum and cyst fluid of mouse models

Upon infection of the host with echinococcal larvae, immune cells are activated and release substantial amounts of reactive oxygen species (ROS) (Wen et al., 2020). ROS stimulates the production of pro-inflammatory cytokines, including IL-6 and TNF-α, which in turn exacerbate the inflammatory response and further promote ROS generation. This interplay creates a positive feedback loop that amplifies both oxidative stress and inflammation (Mahmoud et al., 2024; McElvaney et al., 2021). Conversely, IL-10, an anti-inflammatory cytokine, plays a critical role in suppressing Th1-mediated immune responses. A reduction in IL-10 levels suggests that the Th1 response is heightened, thereby enhancing the host’s anti-echinococcal activity (Shirgholami et al., 2021). Moreover, the Th1-associated cytokine IFN-γ contributes to host defense by enhancing the phagocytic and parasiticidal functions of macrophages and promoting the cytotoxic activity of T cells and natural killer cells. These immune mechanisms collectively facilitate the clearance of echinococcal larvae (Zhang Y et al., 2021).

**FIGURE 7:**
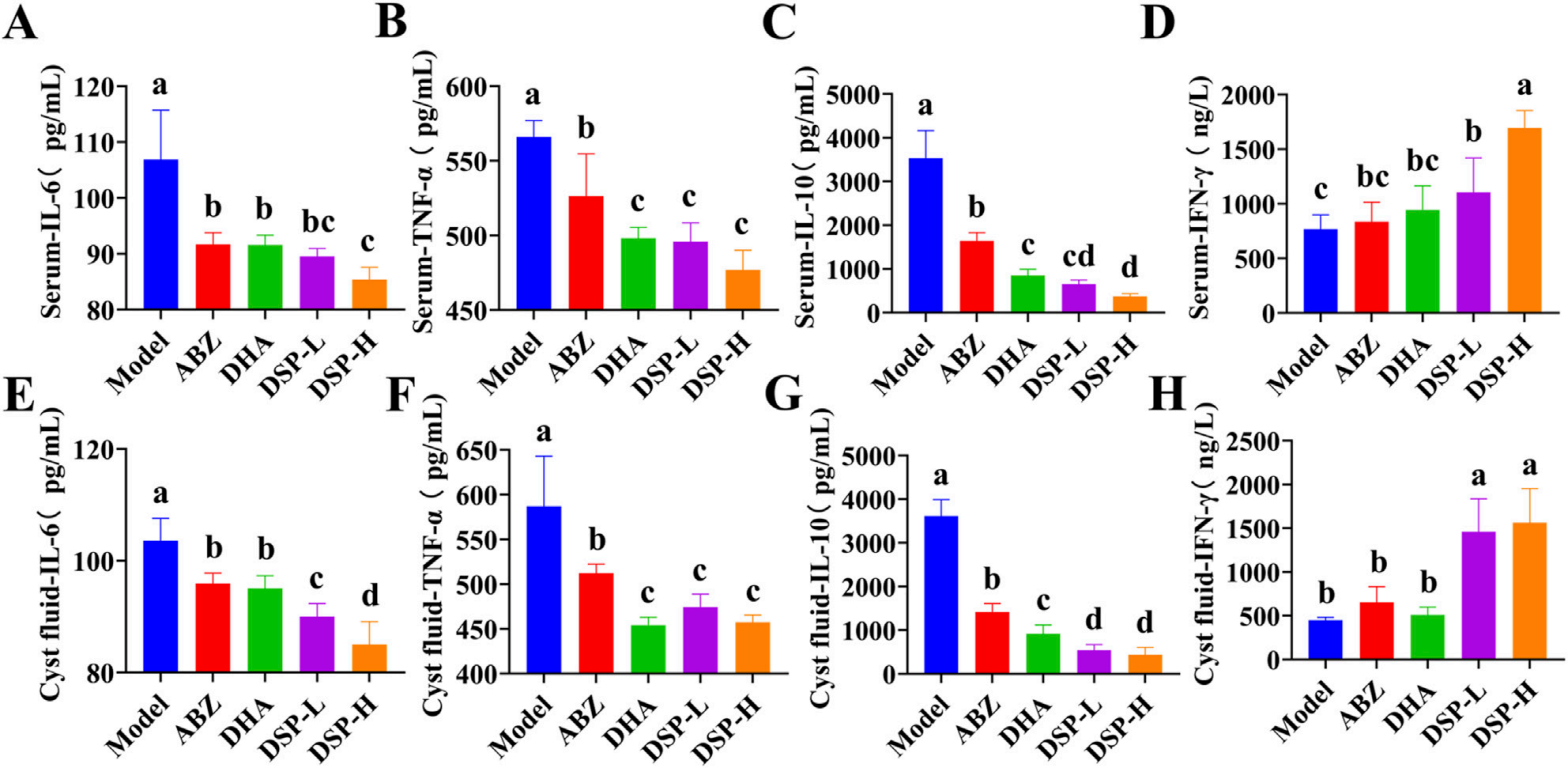
Serum and cyst fluid cytokine assays. **(A)** Serum IL-6 level (n = 5), **(B)** Serum TNF-α level (n = 5), **(C)** Serum IL-10 level (n = 5), **(D)** Serum IFN-γ(n = 5), **(E)** Cyst fiuid IL-6 level (n = 5), **(F)** Cyst fiuid TNF-α level (n = 5), **(G)** Cyst fiuid IL-10 level (n = 5), **(H)** Cyst fiuid IFN-γ level (n = 5). Lowercase letters a, b, c, and d indicate significant differences (*P* < 0.05).

As illustrated in Figures 7A–C,E–G, the serum and cyst fluid levels of pro-inflammatory cytokines IL-6 and TNF-α, as well as anti-inflammatory cytokine IL-10, were significantly reduced in the ABZ, DHA, DSP-L, and DSP-H treatment groups as compared to the model group (*P* < 0.05). Notably, the DSP-H group demonstrated significantly lower levels of IL-6 and IL-10 compared to the ABZ and DHA groups (*P* < 0.05). As illustrated in Figures 7D,H, the serum and cyst fluid level of IFN-γ was significantly elevated in the ABZ, DHA, DSP-L, and DSP-H groups relative to the Model group, with the DSP-H group showing a particular increase compared to both the Model and ABZ groups (*P* < 0.05). Collectively, these findings suggest that ABZ, DHA, DSP-L, and DSP-H can effectively mitigate the inflammatory response and help maintain immune homeostasis, with DSP-H demonstrating a particularly pronounced effect in reducing inflammation and sustaining immune balance.

### 3.10 Anti-*E. granulosus* effect of DSP nanoparticles *in vitro*

**FIGURE 8:**
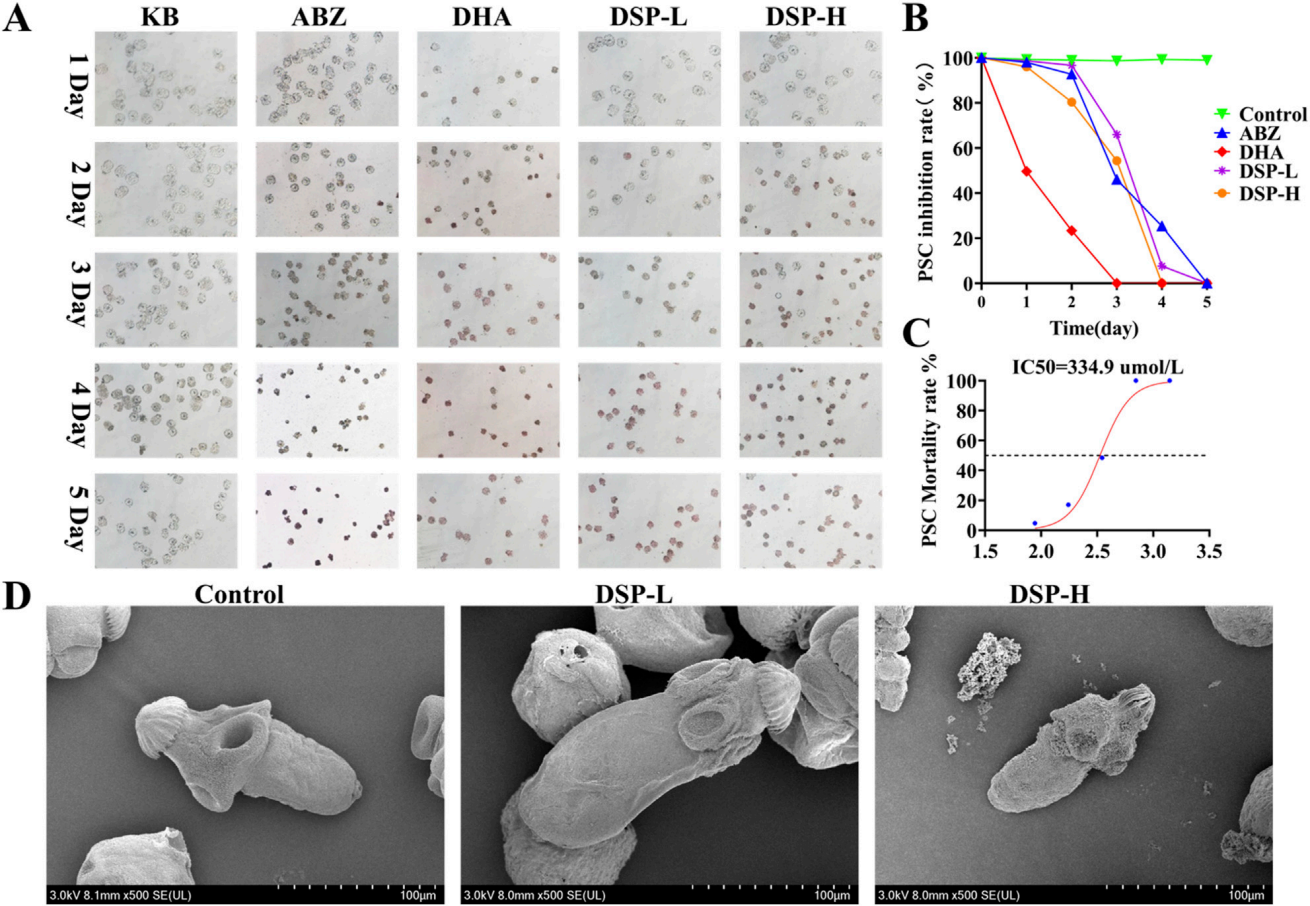
Anti- *E. granulosus* effect of DSP *in vitro*. **(A)** *In vitro* inhibitory effect of the drug on *E. granulosus* (n = 3), **(B)** Survival rate of the drug against *E. granulosus* (n = 3). **(C)** IC_50_ value of DSP nanoparticles against *E. granulosus* (n = 3). **(D)** SEM images of *E. granulosus* in the blank and DSP nanoparticles-treated groups.

In *in-vitro* experiments, the efficacy of drug-induced lethality was evaluated using staining techniques and motility assays. SEM provided direct visualization of the morphological alterations in *E. granulosus* protoscoleces before and after drug treatment (Gao et al., 2022). As illustrated in Figures 8A,B, after 7 days of treatment, the protoscoleces in the blank control group retained a normal and intact structure, characterized by clearly visible calcium granules and a high eosin reaction rate. In the ABZ group, no mortality was observed on day 1, partial mortality occurred on day 3, with complete lethality achieved between days 4 and 5. In contrast, the DHA group exhibited complete mortality by day 3. The DSP-L group reached complete mortality on day 4, while the DSP-H group achieved this by day 3. As illustrated in Figure 8C, the IC_50_ value, which represents the half-maximal inhibitory concentration of the drug against protoscoleces, was determined to be 350 μmol/L. Figure 8D demonstrates that in the control group, the surface structure of the protoscoleces remained smooth, characterized by smooth outer walls, arranged hooklets, and a transparent cyst structure, with no visible depression or structural damage. In contrast, protoscoleces in the DSP-L and DSP-H groups exhibited significant surface damage, including folds and depressions in the outer cyst. Hooklets were detached or missing, and their structures appeared blurred or disrupted. These alterations were more severe in the DSP-H group compared to the DSP-L group. These findings indicate that DSP nanoparticles significantly reduce the survival rate of *E. granulosus* protoscoleces and disrupt the integrity of the outer cyst wall, leading to dehydration or collapse of the internal structure, ultimately resulting in shrinkage of the parasite.

### 3.11 Transcriptomic analysis of DSP anti-*E. granulosus*

**FIGURE 9:**
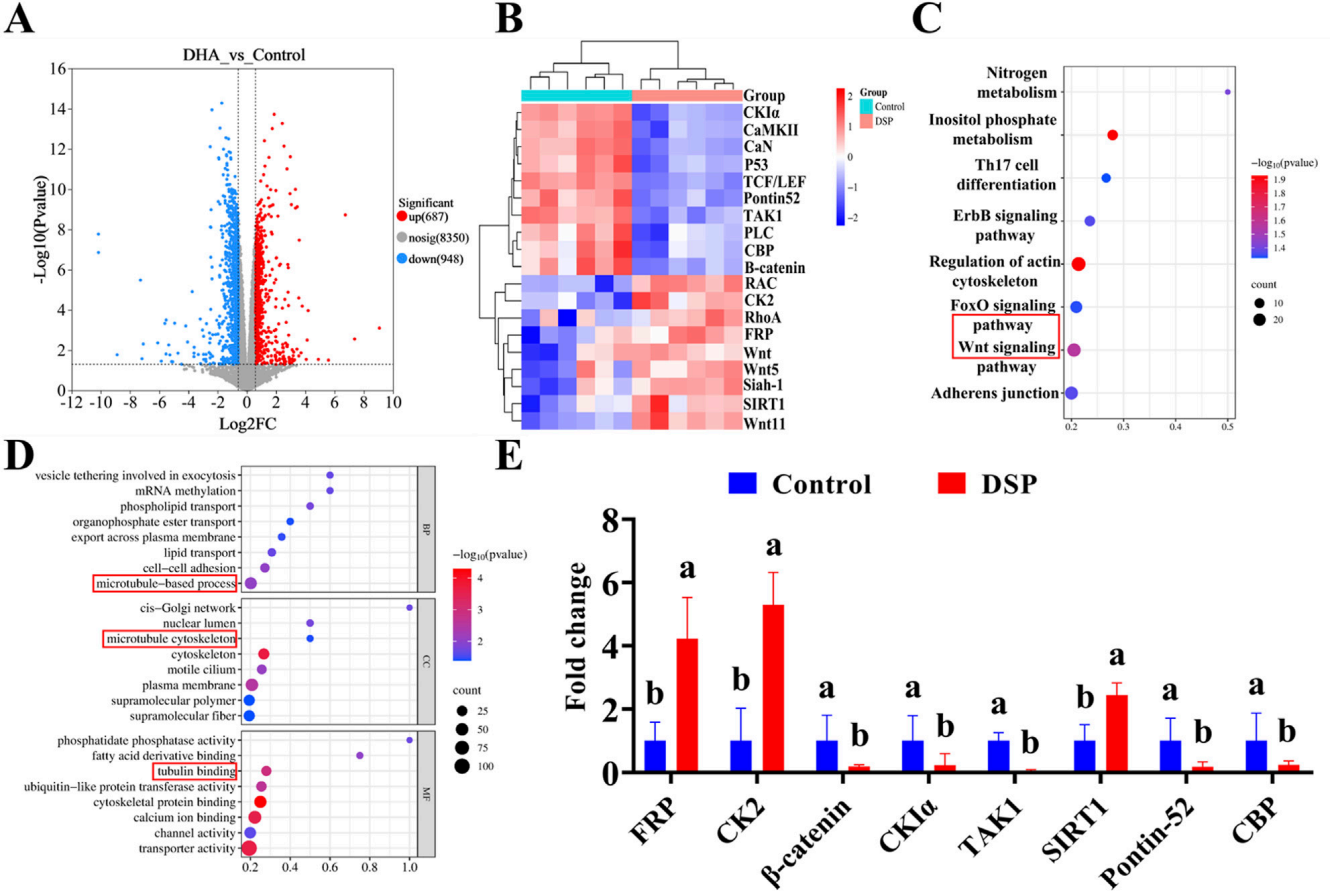
Transcriptomic analysis of DSP anti- *E. granulosus*. **(A)** Differential gene expression, **(B)** Cluster heatmap, **(C)** KEGG pathway, **(D)** GO enrichment analysis, **(E)** qPCR was used to verify the key genes (n = 3). Lowercase letters a, b, indicate significant differences (*P* < 0.05).

Transcriptomics involves the comprehensive analysis of gene expression at the RNA level, providing critical insight into cellular response to various treatments. A volcano plot is a commonly used visualization tool that effectively illustrates gene expression differences and their statistical significance (Xu et al., 2022). As shown in Figure 9A, volcano plot analysis revealed a significant number of differentially expressed genes in the DSP nanoparticles group relative to the control group following protoscolex treatment. Specifically, 687 genes were significantly upregulated, while 948 genes were downregulated. The experimental findings demonstrated that DSP nanoparticles exerted anti-echinococcal effects through transcriptional modulation, as evidenced by significant alterations in gene expression profiles observed in *E. granulosus* protoscoleces following nanoparticle treatment. Cluster heatmaps categorize differentially expressed genes based on similarities in expression pattern, facilitating the identification of potential co-regulated networks or functional modules (Fan et al., 2020). As illustrated in Figure 9B, the DSP nanoparticles-treated group exhibited notable alterations in gene expression compared to the control group. Specifically, genes such as CKIα, CaMKII, CaN, P53, TCF/LEF, Pontin52, TAK1, PLC, CBP, and β-catenin were downregulated, while RAC, CK2, RhoA, FRP, Wnt, Wnt5, Siah-1, SIRT1, and Wnt11 were upregulated. Notably, among these genes, Wnt, FRP, CK2, β-catenin, CKIα, TAK1, TCF/LEF, Pontin52, and CBP are key components of the Wnt/β-catenin signaling pathway (Bauer et al., 2000; Song et al., 2024; Yang et al., 2018). Additionally, Wnt11, RAC, and RhoA are associated with the Wnt/PCP pathway (Choi et al., 2024), while Wnt5, PLC, CaN, and CaMKII are involved in the Wnt/Ca^2+^ pathway (Zuo X et al., 2022).

The Kyoto Encyclopedia of Genes and Genomes (KEGG) analysis provides insight into gene interactions within specific pathways, whereas Gene Ontology (GO) enrichment analysis identifies significantly enriched biological processes to determine the primary functional roles of target genes (Baldwin and Phillips, 2014). As illustrated in Figure 9C, the integration of volcano plot and cluster heatmap analyses, coupled with KEGG pathway analysis, indicates that DSP nanoparticles may inhibit *E. granulosus* multilocularis by activating genes within the canonical Wnt signaling pathway. Previous studies highlighted the pivotal role of the Wnt signaling pathway in *E. multilocularis* development, particularly in the formation and maintenance of essential structures such as the cyst wall and the primitive body cavity (Wang Z et al., 2016; Xiao et al., 2022).

As illustrated in Figure 9D, Gene Ontology (GO) enrichment analysis further supports these findings by revealing significant enrichment in microtubule-based processes across the three categories: biological process involving microtubule-based process, cellular component (CC) category highlighting the microtubule cytoskeleton, and the molecular function (MF) category involves tubulin binding, all of which are intricately linked to the Wnt signaling pathway. Microtubules are known to regulate the canonical Wnt pathway by modulating β-catenin transport, degradation, and signal activation (Ding et al., 2018). Moreover, the stability of the microtubule cytoskeleton modulates Wnt signaling, while tubulin-binding proteins play a pivotal role in Wnt signal transduction through their interaction with β-catenin (Kikuchi and Arata, 2024). To further verify the role of the Wnt pathway, quantitative analysis of key genes in this pathway was performed by Q-PCR. As illustrated in Figure 9E, qPCR validation confirmed that, compared to the control group, the expression level of β-catenin, TAK1, CKIα, Pontin52, and CBP were markedly downregulated in the DSP nanoparticles group (*P* < 0.05), while FRP, CK2, and SIRT1 were significantly upregulated (*P* < 0.05). These findings collectively suggest that DSP nanoparticles may exert potent anti-*Echinococcus granulosus* effects by modulating the Wnt/β-catenin signaling pathway.

## 4 Discussion

Cystic echinococcosis, caused by the larval form of *E. granulosus*, is a severe parasitic disease marked by hydatid cyst formation in host organs, especially the liver, resulting in structural damage and compromised hepatic function. Albendazole is the primary chemotherapeutic agent; however, its clinical effectiveness is constrained by poor solubility and low bioavailability. Recent research indicates that artemisinin derivatives, like DHA, have strong activity against *E. granulosus*. In this study, we successfully developed a drug delivery system (DSP) with a high DHA loading capacity and excellent physicochemical stability. These DSP nanoparticles demonstrated significant liver-targeting capabilities, reduced hydatid cyst formation, and improved liver injury *in vivo*. Mechanistically, their antiparasitic effects on *E. granulosus* protoscoleces may partly occur through the modulation of the Wnt signaling pathway.

Prolonged drug retention in hepatic lesion areas is a critical factor for enhancing therapeutic efficacy in the treatment of hepatic alveolar *E. granulosus*. However, the current DSP nanoparticle-based drug delivery system has significant limitations, particularly its short hepatic retention time. Although promising therapeutic outcomes of DSP nanoparticles have been observed in animal models, their pharmacokinetics, optimal dosing strategies, safety profile, and clinical efficacy in humans require thorough investigation. Furthermore, while the anti-*E. granulosus* effects of this drug are mediated via the Wnt signaling pathway, the complexity of this pathway *in vivo*, involving multiple cascades and regulatory factors, suggests that its precise mechanistic role may not yet be fully elucidated. This knowledge gap could hinder a comprehensive understanding of the drug’s mechanism of action and impede further pharmacological optimization. Future studies should prioritize the detailed characterization of these signaling pathways to accelerate the clinical translation of DSP nanomedicine-based therapies.

## 5 Conclusion

Targeted nano-drugs specifically focus on the liver, significantly improving treatment efficacy against cystic echinococcosis by ensuring precise delivery and sustained-release technology, while overcoming the limitations associated with traditional medications. DHA shows strong antiparasitic effects against *E. granulosus*. In this study, we developed DSP, nanoparticles composed of DHA-loaded PLGA and STC, which demonstrate effective liver-targeting abilities. These nanoparticles effectively reduced the proliferation of hydatid cysts and alleviated liver dysfunction, exerting anti-echinococcal effects through the Wnt signaling pathway. Our results emphasize DSP nanoparticles as a promising targeted nanotherapeutic option for hepatic echinococcosis, enhancing both the precision and safety of anti-echinococcal treatments. Additionally, investigations into the mechanisms of nanoparticle action provide valuable insights that can advance the use of nano-drugs in parasitology and clarify the therapeutic mechanisms involved. This nanomedicine adopts a dual therapeutic approach, combining the targeted elimination of *E. granulosus* with simultaneous liver tissue repair, presenting new opportunities for managing cystic echinococcosis.

## Data Availability

The datasets presented in this study can be found in online repositories. The names of the repository/repositories and accession number(s) can be found below: https://www.ncbi.nlm.nih.gov/, PRJNA1233845.
