# Supplementary material for: Dihydroartemisinin-sodium taurocholate-PLGA nanoparticles: a novel therapeutic approach against cystic echinococcosis
Source: Front Pharmacol. 2025 Jun 11;16:1600525. doi: 10.3389/fphar.2025.1600525 (PMC12187603; doi:10.3389/fphar.2025.1600525)
Supplement: Supplementary file 1 [file DataSheet1.docx]

**Preparation and Anti-*****Echinococcus granulosus* Activity of Dihydroartemisinin-Sodium Taurocholate-PLGA Nanoparticles**

As illustrated in Figure S1A-C, the contour lines for DHA/PLGA, STC, and UP exhibited an oval shape, with significant differences between the major and minor axes. As illustrated in Figure S1D-F, the planar graph demonstrates the optimal region and most favorable combination of the three factors: DHA/PLGA, STC, and UP. These results suggest that the encapsulation efficiency of DHA initially increases but subsequently decreases as the STC concentration, DHA/PLGA ratio, and UP power are progressively increased. Notably, the combination of an STC concentration of 40 mg/mL, a DHA/PLGA ratio of 1:10, and a UP power of 80 W yields the most effective response.


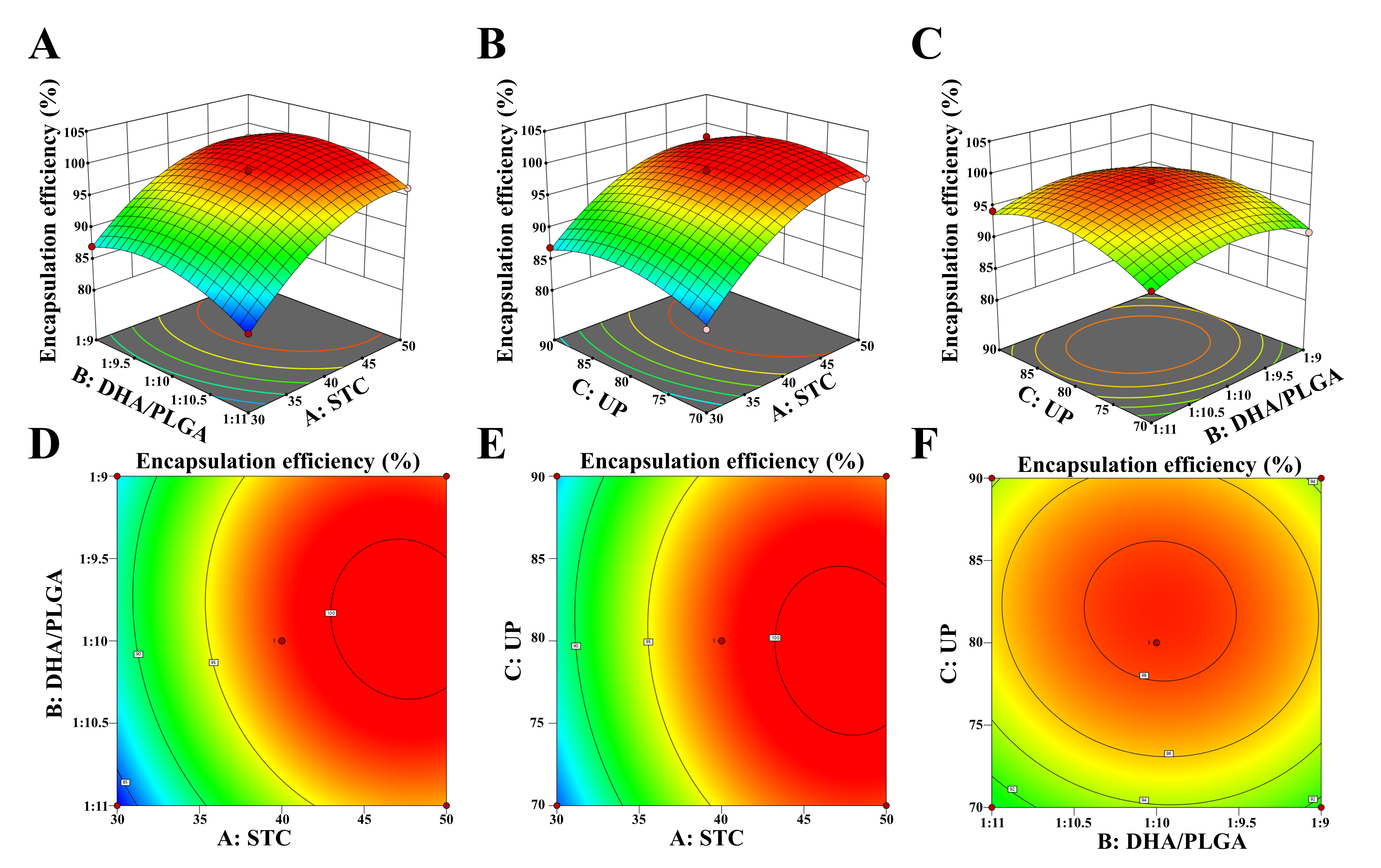


Figure S1: (A) STC and DHA/PLGA Response Surface Contour Plot, (B) STC and UP Response Surface Contour Plot, (C) DHA/PLGA and UP Response Surface Contour Plot, (D) STC and DHA/PLGA 2D Response Surface Plot, (E) STC and UP 2D Response Surface Plot, (F) DHA/PLGA and UP 2D Response Surface Plot.

As illustrated in Figure S2, a standard curve (S2A) was established and the peak area of the unencapsulated DHA content in DSP was calculated by liquid chromatography (HPLC) to obtain the encapsulation efficiency of DHA in DSP. The peak time of DHA (S2B) is indicated by the red dot.





Figure S2: DHA standard curve and DSP HPLC chromatogram.

As illustrated in S3, the sections of liver, intestine, heart, kidney, and spleen from mice stained with HE showed no significant pathological changes, indicating that DSP had no adverse effects on these organs and exhibited good safety.


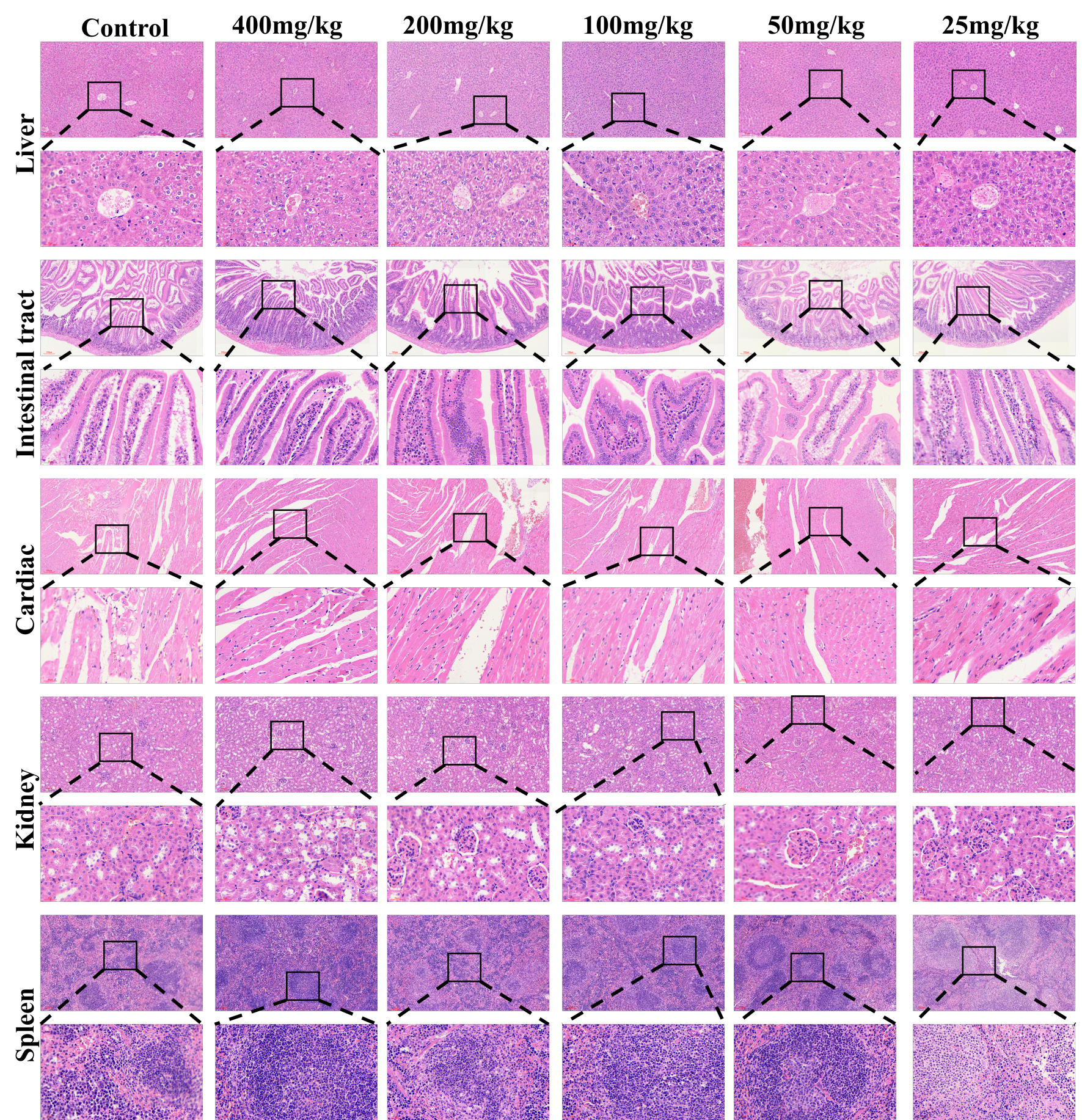


Figure S3: Representative hematoxylin and eosin (HE) stained sections of the liver, intestine, heart, kidney, and spleen from control mice and mice treated with DSP at doses of 400, 200, 100, 50, and 25 mg/kg.
